# Molecular Characterization of Dengue Virus Strains from the 2019–2020 Epidemic in Hanoi, Vietnam

**DOI:** 10.3390/microorganisms11051267

**Published:** 2023-05-11

**Authors:** Juthamas Phadungsombat, Huong Thi Thu Vu, Quynh Thi Nguyen, Ha Thi Van Nguyen, Ha Thi Nhu Nguyen, Bich Thi Dang, Emi E. Nakayama, Azumi Ishizaki, Hiroshi Ichimura, Tatsuo Shioda, Thach Ngoc Pham

**Affiliations:** 1 Department of Viral Infections, Research Institute for Microbial Diseases, Osaka University, Osaka 565-0871, Japan; juthamas@biken.osaka-u.ac.jp (J.P.); emien@biken.osaka-u.ac.jp (E.E.N.); 2 National Hospital for Tropical Disease, Hanoi 100000, Vietnam; huongvu13@gmail.com (H.T.T.V.); vumanhhungha@gmail.com (H.T.V.N.); nhuha.niitd@yahoo.com (H.T.N.N.); bich.vaac@gmail.com (B.T.D.); phamngocthachnhtd@gmail.com (T.N.P.); 3 Department of Viral infection and International Health, Graduate School of Medical Science, Kanazawa University, Kanazawa 920-8640, Japan; rubynguyen.hmu@gmail.com (Q.T.N.); aishizak@staff.kanazawa-u.ac.jp (A.I.); ichimura@med.kanazawa-u.ac.jp (H.I.)

**Keywords:** DENV-1, DENV-2, DENV genotype, phylogenetic tree, Vietnam, Hanoi

## Abstract

Dengue virus (DENV), which has circulated in Vietnam for several decades, has multiple serotypes and genotypes. A 2019 dengue outbreak resulted in a larger number of cases than any other outbreak. We conducted a molecular characterization using samples collected in 2019–2020 from dengue patients in Hanoi and nearby cities located in northern Vietnam. The circulating serotypes were DENV-1 (25%, *n* = 22) and DENV-2 (73%, *n* = 64). Phylogenetic analyses revealed that all DENV-1 (*n* = 13) were genotype I and clustered to local strains circulating during the previous outbreak in the 2017, whereas DENV-2 consisted of two genotypes: Asian-I (*n* = 5), related to local strains from 2006–2022, and cosmopolitan (*n* = 18), the predominant genotype in this epidemic. The current cosmopolitan virus was identified as having an Asian-Pacific lineage. The virus was closely related to strains in other recent outbreaks in Southeast Asian countries and China. Multiple introductions occurred in 2016–2017, which were possibly from maritime Southeast Asia (Indonesia, Singapore, and Malaysia), mainland Southeast Asia (Cambodia and Thailand), or China, rather than from an expansion of localized Vietnamese cosmopolitan strains that were previously detected in the 2000s. We also analyzed the genetic relationship between Vietnam’s cosmopolitan strain and recent global strains reported from Asia, Oceania, Africa, and South America. This analysis revealed that viruses of Asian-Pacific lineage are not restricted to Asia but have spread to Peru and Brazil in South America.

## 1. Introduction

Dengue is a mosquito-borne disease transmitted to humans via the bite of infected mosquitos and commonly occurs in over 100 tropical and subtropical countries. Clinically, dengue cases vary in severity from mild to fatal. The World Health Organization (WHO) categorizes the disease as dengue (with or without warning signs) and severe dengue [1]. Symptoms, such as high fever, severe headache, muscle and joint pain, nausea, and rash, manifest for 2–7 days, and warning signs of severe disease include severe abdominal pain, persistent vomiting, rapid breathing, bleeding gums, and nosebleed [2]. The incidence of dengue cases reported to the WHO increased from approximately 500,000 cases in 2009 to 5 million cases in 2019 [2], resulting in significant global human and economic impacts.

The causative agent of dengue fever is dengue virus (DENV) (genus *Flavivirus*, family *Flaviviridae*). DENVs can be classified into four serotypes (DENV-1, DENV-2, DENV-3, and DENV-4). In primary DENV infection, long-term host immunity is specifically generated against the actual serotype, with short-term protection against other serotypes. Subsequent secondary infection with a different serotype can lead to antibody-dependent enhancement, increasing the risk of severe dengue [3]. Within the dengue serotypes, 4–6 genotypes have been genetically defined [4]. Serotype/genotype replacement has occurred concurrently with the increase in the number of patients [5].

Vietnam, located in Southeast Asia and bordered by Laos, Cambodia, and China, is a hyperendemic country for dengue. Periodic and cyclic DENV circulation patterns have occurred in Vietnam, but DENV-1 and -2 are the most common serotypes [5,6]. In 2019, the highest recorded number of dengue cases was reported worldwide, particularly affecting Asia, with 420,000, 320,000, 131,000, and 101,000 cases reported in the Philippines, Vietnam, Malaysia, and Bangladesh, respectively [2]. The previous molecular epidemiological studies in Vietnam described an increasing in the dengue incidence associated with the changes in DENV-2 genotypes [5]. Recently, the 2017 outbreak was shown to be caused by local DENV-1 strains [7]. To date, the DENV characteristics responsible for the more recent outbreak in the 2019–2020 season have not been well elucidated. Therefore, we conducted a molecular characterization of DENV isolates from patient samples collected between 2019 and 2020 in Hanoi, the capital city of Vietnam located in the northern part of the country, and nearby provincial cities. We also present clinical data regarding these cases.

## 2. Materials and Methods

### 2.1. Patients, Sample Processing, and Laboratory Testing

Clinical samples were collected from patients with suspected dengue infection who were aged 18 years or older and had a fever (within 5 days of the study). Samples were collected according to the usual diagnostic procedure of the National Hospital for Tropical Disease (NHTD), Hanoi, Vietnam. Ethical Approval was obtained from the Ethics Committee of the NHTD, and all participants gave informed consent. Serum from participating patients was locally analyzed by Dengue Duo (SD Bioline, Seoul, Republic of Korea) to detect the NS1 antigen and dengue-specific antibodies (IgM and IgG). RNA extraction and dengue serotyping were conducted for all NS1-positive serum samples. Viral RNA was extracted from 140 μL of each serum sample using a QIAamp viral mini kit (Qiagen, Hilden, Germany), and 5 μL was used to determine the dengue serotype by multiplex real-time RT-PCR using a Genesig kit (Genesig, Chandler’s Ford, UK).

### 2.2. Virus Isolation

Thirty microliters of serotyping-positive patient serum was inoculated in C6/36, an *Aedes albopictus* cell line, as described previously [8]. A total of 140 μL of culture supernatant was harvested on days 7 and 14 post-infection, and DENV RNA was extracted and analyzed using real-time RT-PCR [9].

### 2.3. Envelope Nested RT-PCR and Whole-Genome RT-PCR

To amplify the DENV envelope region, 5 μL of extracted RNA from patient serum was primarily reverse transcribed and amplified (1°PCR), targeting the first and second halves of the envelope region using a One-step RT-PCR kit (Qiagen) with primers covering the envelope region (Table S1). The PCR master mix was prepared according to the manufacturer’s protocol at a final primer concentration of 0.6 μM. To increase the DNA concentration, the primary PCR product was subjected to secondary PCR (2°PCR) using GXL polymerase (Takara, Shiga, Japan) and primers (Table S1) that amplified the first and second halves of the envelope region product.

To amplify the whole DENV genome, 5.5 μL of extracted RNA of each isolate was used to synthesize cDNA with two primers specific to the first (5′half) and second (3′half) halves of the genome using SuperScript IV (Invitrogen, Vilnius, Lithuania). The RT reaction mixture was prepared according to the manufacturer’s protocol. The 5′half and 3′half cDNAs were amplified using corresponding primers [8] and GXL polymerase (Takara, Shiga, Japan).

The amplified products were examined by gel red staining (Biotium, Fremont, CA, USA) and agarose gel electrophoresis at 100 volts in TBE buffer. The target band was purified using Nucleospin kit (MACHEREY-NAGEL, Düren, Germany). DNA concentration was quantified using either a Nanodrop (Thermo Fisher Scientific, Waltham, MA, USA) or Qubit system (Invitrogen, Eugene, OR, USA).

### 2.4. Envelope and Whole-Genome Sequencing

The nucleotide sequence of the genome region encoding the envelope was determined by Sanger sequencing using an ABI sequencer. The sequencing reactions were prepared using an ABI Prism BigDye Terminator Cycle Sequencing Ready Reaction kit, version 3.1 (Applied Biosystems, Vilnius, Lithuania). Briefly, 2 μL of DNA template was mixed with 2 μL of reaction mix, 1 μL of 5× sequencing buffer, 1.6 μL of specific primer (Table S1), and 3.4 μL of nuclease-free water. The amplification cycle conditions were programmed according to the manufacturer’s protocol.

The whole genome sequences of DENV isolates obtained in the present study were determined by next-generation sequencing (NGS) on a Miseq platform (Illumina, San Diego, CA, USA). Preparation of the NGS library using NexteraXT (Illumina, San Diego, CA, USA) was described previously [8]. The FASTQ results were examined using CLC Genomics Workbench software, version 21 (CLC Bio, QIAGEN, Valencia, CA, USA). Whole-genome assembly was conducted using Map read reference command (DENV-1 Mochizuki AB074760.1 and DENV-2 16681 NC_001474.2 were used as reference strains).

All newly obtained sequences of the envelope and the whole genome were deposited in GenBank with accession numbers OQ832560-OQ832594 and OQ832609-OQ832627, respectively.

### 2.5. Phylogenetic and Genetic Diversity Analyses

DENV sequences analyzed in the present study were retrieved by February 2023 from GenBank and The Bacterial and Viral Bioinformatics Resource Center (BV-BRC) (https://www.bv-brc.org/ (accessed on 15 February 2023)) [10] and combined with the new Vietnam sequences (Table S2). Alignment and translation were conducted in AliView v1.26 [11]. The maximum-likelihood (ML) tree and model selection were estimated in IQ-TREE for DENV-1 and DENV-2 using the envelope region [12]. The molecular clock phylogeny of DENV-2 cosmopolitan based on the envelope region was estimated using BEAST, v1.10 [13]. The temporal signal dataset was examined by root-to-tip using Tempest and showed R^2^ of 0.87 (Figure S1) [14]. Triplicate BEAST runs using settings for the SRD06 and Skygrid coalescent model with either a strict clock or uncorrelated clock model were employed for >50 million Markov Chain Monte Carlo cycles with 5 million cycle burn-ins, and marginal-likelihood estimation was performed using path sampling and stepping stone sampling analyses, which suggested that the strict clock was the best fit for this analysis (Table S3) [15,16]. The run convergence was accessed in Tracer, v1.7.1 [17]. Triplicate runs obtaining an effective sample size of >200 were combined in Logcombiner, and the maximum clade credibility (MCC) phylogeny was generated using TreeAnnotator and visualized in Figtree, v1.4.4.

To analyze amino acid polymorphism in Hanoi DENVs, the coding sequences were compared to previous Vietnam sequences available in GenBank. The amino acid position was annotated corresponding to the reference viruses: NC_001477.1, NC_001274.2, and GQ398263 for DENV-1 genotype I, DENV-2 genotype Asian-I, and DENV-2 genotype cosmopolitan, respectively.

### 2.6. Statistical Tests

Differences in categorical and continuous variables were statistically evaluated by Fisher’s Exact test and Mann–Whitney U test, respectively. All the statistical calculations were performed by GraphPad Prism (GraphPad Prism Software, Boston, MA, USA).

## 3. Results

### 3.1. DENVs in Hanoi, Vietnam, in 2019–2020

**Figure 1:**
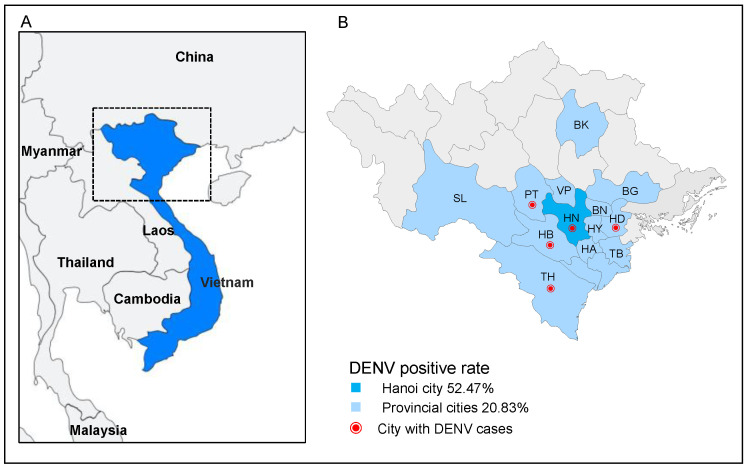
Map of Vietnam showing sample sites and DENV-positivity rates. (**A**) Vietnam is highlighted in blue. (**B**) Map of northern Vietnam; Hanoi (HN) is highlighted in blue. The provincial cities are highlighted in light blue and shown as follows: Hoa Bihn (HB), Phu Tho (PT), Vinh Phuc (VP), Bac Ninh (BN), Hung Yen (HY), Hai Dung (HD), Ha Nam (HA), Thai Binh (TB), Bac Giang (BG), Bac Kan (BK), Son La (SL), and Thanh Hoa (TH). Cities with DENV cases are marked on the map with a red dot.

Between October and December 2019 and September and December 2020, a total of 266 DENV clinical suspected samples were collected at the NHTD (2019, *n* = 30; 2020, *n* = 103), with 201 patient serum samples from Hanoi, the capital city, and 24 from nearby provinces (Hoa Bihn, Phu Tho, Vinh Phuc, Bac Ninh, Hung Yen, Hai Dung, Ha Nam, Thai Binh, Bac Giang, Bac Kan, Son La, and Thanh Hoa) in northern Vietnam (Figure 1); no data were available for 41 serum samples. Of these samples, 133 were DENV NS1–positive (49.61%), 7 were DENV IgM–positive (2.71%), and 11 were DENV IgG-positive samples (4.26%). The DENV-positivity rate was 52.47% and 20.83% for Hanoi city and the provincial cities (Hai Duong, Hoa Bihn, Phu Tho, and Thanh Hoa), respectively.

Among the 133 DENV-positive patients, case records were available for some, as described in Table 1.

Among the 88 DENV serotyped cases, 22 (25%), 64 (72.73%), and 2 (2.27%) were identified as DENV-1, DENV-2, and DENV-4, respectively. DENV-2 was the predominant serotype in 2019 and 2020. The demographic and clinical characteristics of DENV-1 and DENV-2 cases are summarized in Table 1, and there was no significant difference in demographic and clinical laboratory data between DENV-1 and DENV-2 cases. Molecular characterization was performed for 36 cases, consisting of 13 DENV-1 and 23 DENV-2 strains, as presented in Table 2. Unfortunately, the DENV-4 sequence was unobtainable due to the low viral load.

### 3.2. Persistence of DENV-1 Genotype I in Vietnam

**Figure 2:**
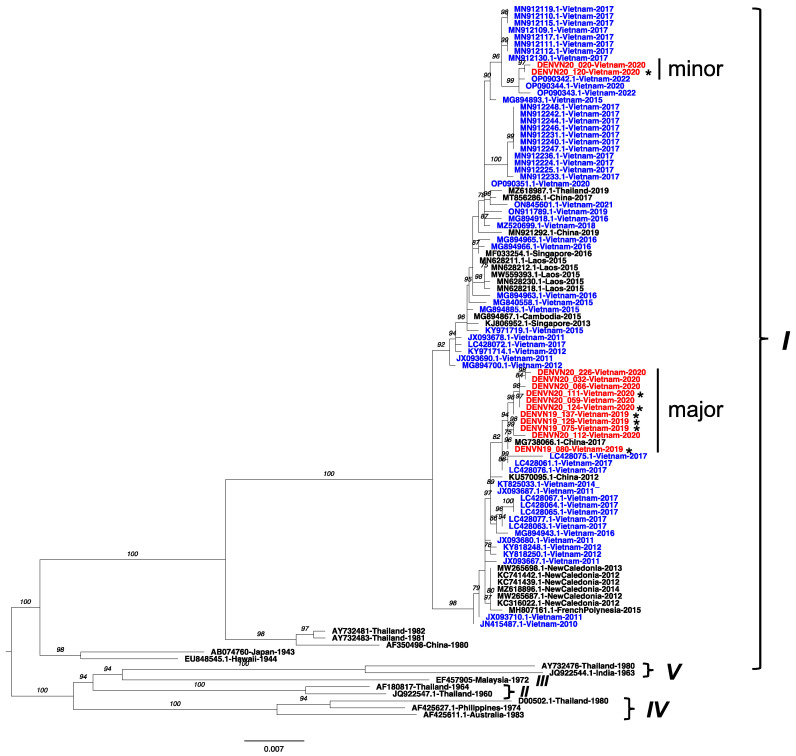
Maximum-likelihood tree of DENV-1 isolates from Vietnam based on the envelope gene. The best nucleotide substitution model was TIM2 + F + G4. The Vietnam strains sequenced in the present study are labeled in red. Of them, the sequences obtained from the whole-genome analysis are marked with an asterisk. Public Vietnam sequences from GenBank are labeled in blue. Genotypes of DENV-1 are indicated to the right. Numbers on branches indicate bootstrap support values (>75%).

The ML tree of the envelope gene region (1485 bp) of the newly obtained sequences was reconstructed together with sequences from GenBank, including DENV-1 genotype references (I–V) and the related sequences from BLASTN searches (Figure 2). All DENV-1 isolates belonged to genotype I and were separated into two clades. First, two DENV-1 isolates (DENVN20_120 and DENVN20_20) were the most closely related to the Vietnam strains collected in 2022 and clustered with Vietnam strains circulating in 2015–2017. Second, the remaining DENV-1 isolates formed the majority (DENVN19_129, DENVN19_137, DENVN19_075, DENVN19_080, DENVN20_124, DENVN20_032, DENVN20_059, DENVN20_066, and DENVN20_226) of those related to the 2017 strains collected in China and Vietnam.

### 3.3. Co-Circulation of the Asian-I and Cosmopolitan Genotype of DENV-2 in Vietnam

**Figure 3:**
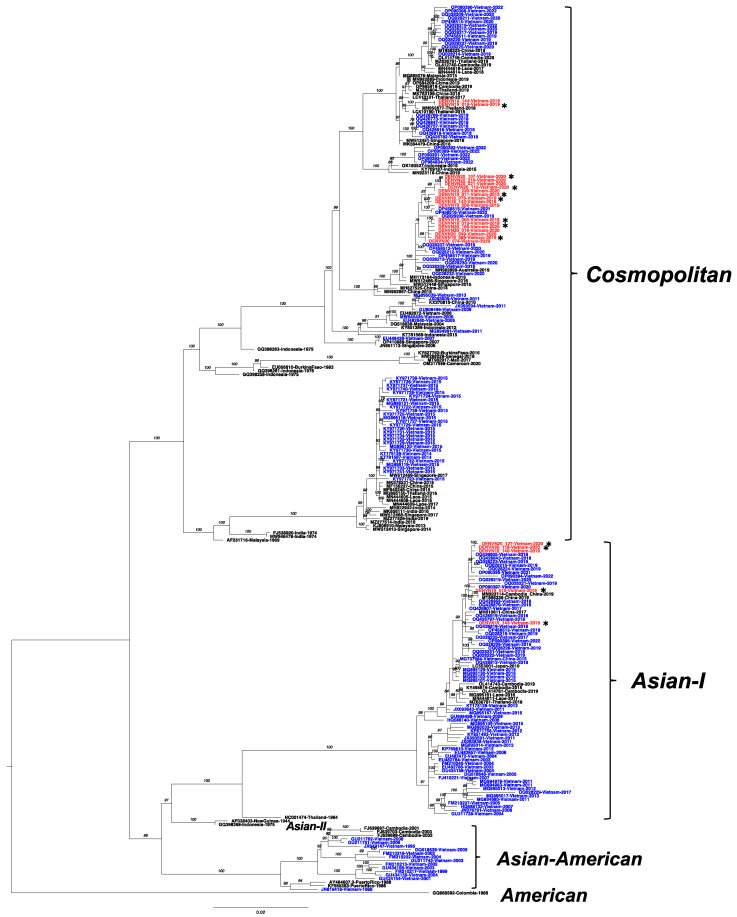
Maximum-likelihood tree of DENV-2 isolates from Vietnam based on the envelope gene. The best nucleotide substitution model was TIM2 + F + I + G4. The Vietnam strains sequenced in the present study are labeled in red. Of them, the sequences obtained from whole genome analysis are marked with an asterisk. Public Vietnam sequences from GenBank are labeled in blue. Genotypes of DENV-2 are indicated to the right. Numbers on branches indicate bootstrap support values (>75%).

The ML tree of the DENV-2 envelope sequences (Figure 3) consisted of the Hanoi 2019–2020 strains reported in the present study, their related sequences obtained by BLASTN searches, other Vietnam strains available in the database, and the genotype representative strains. Five distinct genotypes were separated with branch support (bootstrap 100%). The Hanoi DENV-2 isolates were classified into two genotypes. First, five isolates, including DENVN19_010, DENVN19_140, DENVN19_143, DENVN20_118, and DENVN20_127, belonged to the Asian-I genotype and clustered closely with recently reported viruses isolated from Vietnam in 2017–2022, China in 2017–2019, and Cambodia in 2019. Second, the vast majority (18 strains shown in Table 1) were characterized as the cosmopolitan genotype. Of these cosmopolitan isolates, DENVN19_015 and DENVN19_144, which were collected in 2019, clustered to a small clade with the most-related strain from Thailand collected in 2016 and 2018, whereas the remaining 16 strains (2019–2020) formed a clade together with other Vietnam strains circulating in 2018–2021 and Australia strains collected in 2019.

**Figure 4:**
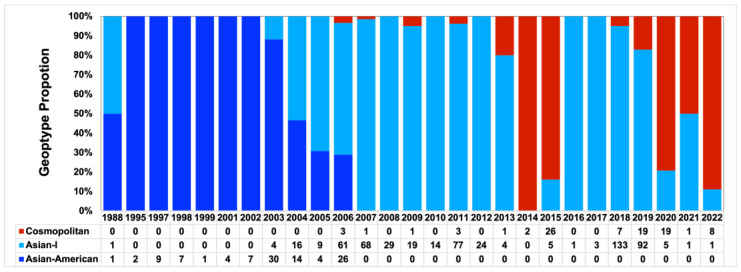
Proportion of the DENV-2 genotype in Vietnam from 1988 to 2022 based on sequences available in GenBank.

Since we observed that the DENV-2 cosmopolitan genotype predominated in the Hanoi epidemic of 2019–2020, we collected public sequences to determine the historical distribution of DENV-2 genotypes in Vietnam from 1988–2022 (Figure 4). The results indicated that there was no circulation of the Asian-American genotype after the middle of the 2000s, but Asian-I was the major genotype over most of the 2005–2013 and 2016–2019 period. Regarding the phylogeny of DENV-2 (Figure 3), Asian-I envelope sequences sampled between 2003 and 2022 formed clusters and descended from earlier Vietnam strains, which is consistent with the larger dataset analysis summarized in Figure S2. This genetic relationship suggested that the Vietnam Asian-I lineage was localized and maintained until the present. On the other hand, the cosmopolitan genotype had multiple introductions. It was detected sporadically in 2006–2007, 2009, and 2011 (Figure 4). Notably, the cosmopolitan genotype dominated for a short time in 2014 and 2015, at 100% and 84%, respectively. The cosmopolitan genotype recently re-emerged between 2018 and 2022 and exhibited a trend of increasing proportion during this period, at 5, 17, 79, 50, and 89%. Based on our results, we observed the cosmopolitan genotype at 75% and 83% of DENV-2 samples in 2019 and 2020, respectively. 

### 3.4. Emergence of DENV-2 Cosmopolitan Lineage C (Asian-Pacific) in Vietnam

**Figure 5:**
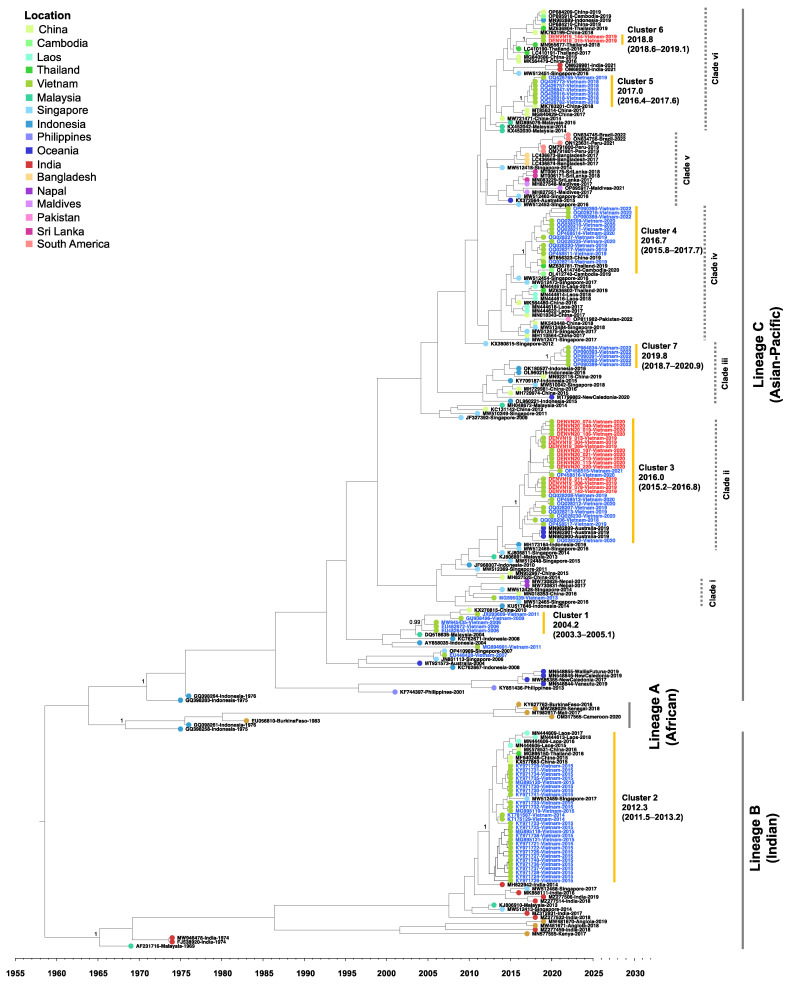
Maximum clade credibility tree of DENV-2 genotype cosmopolitan based on the envelope gene. The Vietnam sequences obtained in the present study and from GenBank are labeled in red and blue, respectively. The grey and dotted grey brackets indicate the lineage and clade, respectively. The yellow bracket indicates Vietnam clusters with the most recent common ancestor (tMRCA) and a 95% highest posterior density interval (HPD). The numbers of posterior probability (PP) support are shown adjacent to the key nodes. The branch tip color corresponds to the location indicated. The timescale in years is shown on the *x*-axis at the bottom.

The time to the most recent common ancestor (tMRCA) of the DENV-2 genotype cosmopolitan was estimated using BEAST, and the result is summarized in an MCC tree in Figure 5. The dataset consisted of the reference strains representing the DENV-2 cosmopolitan lineages previously described [8,18], all available Vietnam and related strains from the database, and strains reported during 2015–2022. Three lineages separated with a posterior of 1, including lineage A (African), lineage B (Indian), and lineage C (Asian-Pacific). Within lineage C (Asian-Pacific), six phylogenetic clades (Clades i–vi) were observed during the 2014–2022 period. The basal strains of each clade were cosmopolitan viruses that circulated prior to mid-2010 in countries in maritime Southeast Asia, including Singapore, Indonesia, and Malaysia, which were cosmopolitan virus hotspots. 

In Figure 5, the Vietnam cosmopolitan viruses formed seven clusters. Cluster 1 strains from 2006–2011 fell into lineage C from China in 2010, and the tMRCA was estimated at 2004.2 (2003.3–2005.1). Cluster 2 strains from 2014–2015 were in lineage B and related to the Indian strain from 2014. The tMRCA was 2012.3 (2011.5–2013.2). Notably, recent Vietnam strains collected during 2018–2022 classified in lineage C separated in clusters 3–7. Cluster 3 was related to the Indonesia strains from 2016, and the tMRCA was estimated at 2016.0 (2015.2–2016.8). The vast majority of our Hanoi strains from 2019–2020 were in this cluster together with the Ho Chi Minh City strain collected in 2018–2020. Furthermore, a Vietnam strain collected in 2020 (OQ028232) in Cluster 3 was most closely related to Australia 2019 strains (MN982899-MN982901). Viruses of Clusters 4, 5, and 6 were most closely related to viruses from China, Thailand, and Cambodia that circulated in 2018–2020, and the tMRCA of these viruses was 2016.7 (2015.8–2017.7), 2017.0 (2016.4–2017.6), and 2018.8 (2018.6–2019.1), respectively. Interestingly, a minor group of Hanoi isolates (DENVN19_015 and DENVN19_144) belonged to Cluster 6 and was distinctly separated from the majority of Hanoi viruses (cluster 3). The ancestral strain of these isolates was closely related to Thailand strains (MN955677 and LC410190). Five Vietnam strains collected in 2022 were in Cluster 7 in which the tMRCA was dated at 2019.8 (2018.7–2020.9). These strains were related to the Indonesia strains from 2016. 

### 3.5. Amino Acid Polymorphisms within Genotypes of Hanoi DENV Strains

**Figure 6:**
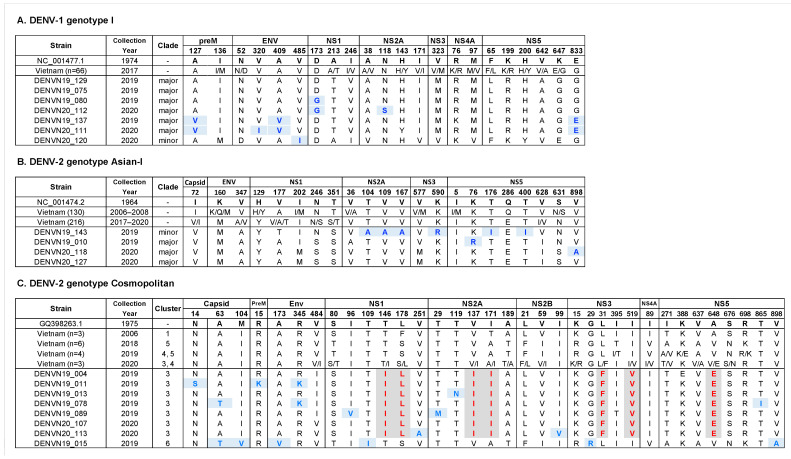
Amino acid polymorphisms within genotypes of Hanoi DENV strains compared to the previous Vietnam strains. The codon numbering with respect to the reference viruses of NC_001477.1, NC_001274.2, and GQ398263.1 for DENV-1 genotype I, DENV-2 genotype Asian-I, and DENV-2 genotype cosmopolitan, respectively. The variations newly observed in the present study and the amino acids specific to certain viral clusters are labeled in blue and red, respectively. The number of previous Vietnam strains is indicated in the parenthesis.

The whole genomes of DENV isolates obtained in the present study were analyzed for amino acid polymorphisms and compared to the previous Vietnam strains (Figure 6). The envelope sequences analyzed above showed complete consistency with the corresponding region in the whole genome sequences for each sample. Among seven strains of DENV-1 genotype I, a total of 22 amino acid differences were observed. DENVN20_111 had the most newly observed mutations, with 4 amino acid differences compared to other strains. For DENV-2 genotype Asian-I strains, 22 different amino acids were observed along the entire coding sequence. The major clade strains had mutations similar to those of the Vietnam strains reported in 2017–2018. Among DENV-2 genotype cosmopolitan viruses, 35 different amino acid substitutions located in both structural and nonstructural genes were observed. The DENVN19_015 isolate, classified in Cluster 6, showed six newly observed mutations, whereas the remaining isolates (DENVN19_004, DENVN19_011, DENVN19_013, DENVN19_078, DENVN19_089, DENVN20_107, and DENVN20_113) that were grouped in Cluster 3 shared the seven specific mutations of NS1-146I, NS1-178L, NS2A-137I, NS2A-171I, NS3-31F, NS3-519V, and NS5-648E. 

## 4. Discussion

In 2019, more than 300,000 dengue cases were reported in Vietnam, an approximately three-fold increase from the previous year [19]. In the present study, DENV patients in Hanoi city and nearby provinces in the northern part of Vietnam during October-December 2019 and September-October 2020 were investigated. The DENV NS1-positivity rate was higher in urban areas, with most DENV cases detected in Hanoi involving no travel history, and four cases occurred in rural areas. These positivity rates indicate that the transmission significantly occurred in the more densely populated areas [20]. Most of the DENV patients in the present study had primary infection with mild illness showing fever, fatigue, and muscle pain as the typical symptoms. A cohort study of hospitalized adult patients in northern Vietnam during 2016–2019 reported that dengue with warning signs in primary infection were found in 33% of patients in the 2017–2018 season and in 17% in the 2018–2019 season. Dengue with warning signs was found more often in secondary infection, at 40% in the 2017–2018 season and at 21% in the 2018–2019 season, while there were few cases of severe dengue [21]. DENV-1 was reported as the primary serotype responsible for the 2017 epidemic [7,22], whereas DENV-2 was the predominant serotype in the 2019–2020 season. These results suggested that the change in serotype influenced the clinical profile seen in the present study.

DENV-1 and DENV-2 were the major serotypes, and very few DENV-4 could be detected in the present study. DENV-2 was the predominant serotype both in 2019 and 2020. In the 2017 epidemic, DENV-1 was the dominant serotype in locations in northern Vietnam such as Hanoi, Ha Nam, and Hai Duong. Subsequently, DENV-1 decreased gradually from 2017 to 2019 perhaps due to the presence of serotype-specific immunity in the human population [7,21,22,23]. Notably, the serotype shift from DENV-1 to DENV-2 noted in our study occurred primarily in northern Vietnam in 2019–2020. However, other regions, such as central Vietnam, had a different predominant type during the period from December 2018 to February 2019, with mostly DENV-4 and a small proportion of DENV-2 [24]. Serotype replacement occurred in southern Vietnam as DENV-2 observed between 2003 and 2006 [5] switched to DENV-1 between 2006 and 2008 [20], whereas in northern Vietnam, and particularly Hanoi, the proportions of DENV-1 and DENV-2 were reportedly equal in 2008 [25]. Dengue transmission is high in Ho Chi Minh City and southern Vietnam, with the annual wave of cases typically peaking in the dry season [26]. Unlike in southern Vietnam, Hanoi has a subtropical climate with four seasons. Dengue transmission in northern Vietnam driven by DENVs from the southern region is usually interrupted by a seasonal bottleneck [27]. However, recent changes in climate have altered the situation. This has led to a greater frequency of spread in the northern region and a higher incidence of dengue, especially between June and November, when the highest temperatures of the year occur [28,29]. Thus, DENV surveillance is required routinely to monitor the new serotype such as DENV-2 in Hanoi through the winter since the serotype shift would link to an increase in the patient number.

We detected DENV-1 as a minor serotype with a single genotype as all 13 DENV-1 isolates were phylogenetically classified as genotype I. Although these viruses clustered in two separate clades, they were related and clustered with local strains that were also collected in 2017, 2019–2020, and 2022. A total of 59,063 dengue fever cases were reported in the 2017 epidemic in northern Vietnam, which was eight times the number of cases reported in 2016 that were associated with DENV-1 genotype I [7,30]. Our results showed that the 2019–2020 DENV-1 genotype I isolates descended from viruses that caused the 2017 epidemic that persisted in this region. Indeed, DENV-1 genotype I has existed in Vietnam since it was first introduced from Thailand during the late 1980s or early 1990s, and it has been imported multiple times from Cambodia in the 2000s [20,27]. DENV-1 genotype I subsequently circulated as the dominant genotype and has now descended into several local clades associated with the latter outbreaks [7].

Among DENV-2 isolates detected in the present study, the cosmopolitan genotype was predominant over the Asian-I genotype during 2019–2020. Genotype replacement has occurred in Vietnam over the last decade. Asian-I, which initially arose as the new type in 2003, subsequently replaced the Asian-American type, the previous local type, between 2003 and 2007 [5]. Since then, the Asian-I type has circulated sustainably until the present [31,32]. However, the co-circulation of Asian-I and cosmopolitan viruses was observed not only in the present study but also in a previous study involving a DENV-infected visitor who had a travel background in Vietnam; that study described the trend in genotype distribution during 2003–2016 [33]. Co-circulation was detected in the single years 2007 and 2011 and continuously between 2013 and 2015. Regarding our DENV-2 phylogenetic tree, Vietnam cosmopolitan viruses were observed in several distinct clades from 2006–2022. The Vietnam 2006–2011 virus (Cluster 1, which was part of a small cluster within lineage C (Asian-Pacific)) and the 2014–2015 virus (Cluster 2, which was within lineage B (Indian)) are both no longer detected. The Vietnam 2018–2022 cosmopolitan viruses were associated with lineage C (Asian-Pacific). In particular, the viruses of clusters 4–6 are closely related to virus strains from bordering countries, including Cambodia, Thailand, and China, that were isolated in 2018–2019 and are probable origins. In contrast, viruses of Clusters 3 and 7 had Indonesia and Singapore strains isolated in 2016 as ancestral strains, indicating that multiple, independent introductions occurred in Vietnam. In the case of Hanoi, most of the Hanoi viruses were in Cluster 3 and were very close to the Ho Chi Minh City strain, the earliest of which was detected in 2018. Moreover, Cluster 3 viruses shared specific amino acid mutations (NS1-146I, NS1-178L, NS2A-137I, NS2A-171I, NS3-31F, NS3-519V, and NS5-648E). This suggests that the virus that spread across southern and northern Vietnam and Ho Chi Minh City was likely the origin of the Hanoi cosmopolitan virus. 

Global cosmopolitan isolates sampled from public databases and isolated in different geographic regions, including Asia, Oceania, Africa, and South America, also fall into lineage C (Asian-Pacific), indicating a global spread with increased DENV cases or outbreaks. Their emergence in southeast Asia, including Thailand 2016–2017, Cambodia 2019–2020, and Vietnam 2018–2022, led to co-circulation with Asian-I, the local genotype [8,34]. In areas of southern Asia, including Nepal 2017, Sri Lanka 2017–2018, Bangladesh 2017–2018, the Maldives 2017–2019, India 2021, and Pakistan 2022 [35,36,37], the cosmopolitan lineage C virus emerged, and this is where the lineage B (Indian) virus had previously circulated. Furthermore, the cosmopolitan lineage C virus spread for the first time to South America, with local transmission reported in Peru in 2019 [38] and Brazil in 2022 [39], both of which were mostly related to the Bangladesh 2017 virus [37]. It is possible that the cosmopolitan lineage C virus successfully adapted to humans, particularly in transmission fitness, resulting in its spread to a wider area and an increase in cases of infection. In the case of Vietnam, more than 300,000 individuals were affected in 2019. Monitoring DENV lineages could provide a better assessment of the epidemic risk as well as advice on resource allocation and guided control actions to minimize transmission intensity.

Multiple DENV clades are often observed in hyperendemic areas. Here, we reported the prevalent clades circulating in Hanoi and the amino acid polymorphisms. Regarding the diversity within DENV genotypes, differences at the nucleotide and amino acid levels were less than 6% and 3%, respectively [40]. DENV has acquired viral fitness or vector competence, and both these mechanisms are known to be associated with viral turnover events such as the persistence or replacement of clades/lineages [4]. For instance, the K160Q/M mutation in the DENV-2 genotype Asian-I viruses that emerged in Vietnam in 2008–2011 caused higher viremia in patients but increased neutralization sensitivity in DENV-2 genotype Asian-American [41]. Interestingly, the K160M mutation was still maintained and detected in the Vietnam Asian-I lineage in the present study. In addition, we have identified new mutations that were suspected to play certain roles in fitness. Their phenotypic effects are still unknown and should be investigated further. Several studies have explained that there was greater replication of the major/dominant clade virus in native mosquitos, thereby enhancing the local transmission. This has been seen in the DENV-1 genotype I clade/lineage shift in Thailand and Cambodia and the DENV-2 Asian-American dominant clade replacement in Nicaragua [42,43,44]. As mentioned above, the newly introduced cosmopolitan lineage C (Asian-pacific) is invading into new areas and subsequently co-circulating with the local genotype, such as Asian I, as shown in the present study. However, the mechanisms of viral evolution are still unclear. Further studies are required to explore the mechanisms or factors involving viral turnovers.

The limitations of this study include the lack of travel history of patients that could illustrate the virus transmission more clearly. Detailed demographic and clinical laboratory data were not available for some patients. The available Vietnam DENV sequences in the database are also limited in some periods. Therefore, more sequence data are critical to monitor the spread of the virus. In conclusion, our study characterized DENV strains associated with a large dengue outbreak in 2019–2020 in northern Vietnam and enhanced our understanding of the recent dynamics of DENV transmission. DENV-1 genotype I and DENV-2 genotype Asian-I are still maintained, but DENV-2 genotype cosmopolitan has re-emerged. We demonstrated that the Hanoi cosmopolitan strain was associated with lineage C (Asian-Pacific) and related to viruses from neighboring countries. In addition, identifying the dengue genotype and estimating the time the outbreak strain emerged are important for determining the origin, routes of transmission, and circulation of DENVs as well as for evaluating vaccine performance and virus control efforts.

## Figures and Tables

**Table 1:** Demographic and clinical characteristics of DENV patients.

	Total DENV (*n* = 133)		DENV-1 (*n* = 22)		DENV-2 (*n* = 64)		*p*-Value
	Frequency	Percentage	Frequency	Percentage	Frequency	Percentage	
Sex							
Male	55	41.35	11	50.00	28	43.75	0.6290 ^b^
Female	51	38.35	8	36.36	22	34.38	>0.9999 ^b^
No data	27	20.30	3	13.64	14	21.88	0.5413 ^b^
Age							
18–29	73	54.89	14	63.64	31	48.44	0.3225 ^b^
30–49	41	30.83	8	36.36	23	35.94	>0.9999 ^b^
>50	13	9.77	0	0.00	8	12.50	0.1071 ^b^
N/A	6	4.51	0	0.00	2	3.13	>0.9999 ^b^
Symptoms ^a^							
Fever	99	98.02	17	94.44	48	97.96	0.4681 ^b^
Fatigue	91	88.35	17	94.44	43	87.76	0.6645 ^b^
Muscle pain	55	53.40	9	50.00	21	42.86	0.7823 ^b^
Joint pain	26	25.24	5	27.78	11	22.45	0.7488 ^b^
Skin rash	7	6.80	2	11.11	3	6.12	0.6051 ^b^
Headache	6	5.83	2	11.11	1	2.04	0.1735 ^b^
Tourniquet	5	4.85	1	5.56	3	6.12	>0.9999 ^b^
Abdomen pain	4	3.88	2	11.11	2	4.08	0.2909 ^b^
Joint swelling	3	2.91	1	5.56	1	2.04	0.4681 ^b^
Vomit	2	1.94	0	0.00	2	4.08	>0.9999 ^b^
Other (gingival bleeding, ascites, and reduction in urination)	3	2.91	0	0.00	3	6.12	0.5581 ^b^
Chemical ^a^	Median	IQR	Median	IQR	Median	IQR	
Platelets (10^3^/μL)	158	126–185	131.00	115.50–194.75	162	129–183	0.2470 ^c^
White blood cells (10^3^/μL)	4.6	3–5.9	3.8	2.98–5.98	4.9	3.15–6.3	0.3337 ^c^
Hemoglobin (g/L)							
Male	148	141–155.5	146	140–155	147	141–153	0.9431 ^c^
Female	131	125–141.25	124	111–145	131	126–134.25	0.1245 ^c^
Hematocrit (%)							
Male	43.3	41.4–45.3	43	41.6–44.9	42.5	40.8–45.3	0.9557 ^c^
Female	38.35	36.75–41.35	36.6	34–43.8	38.45	36.95–39.9	0.2858 ^c^

^a^ Data available for only 18 DENV-1 and 49 DENV-2 cases. ^b^ Fisher’s exact test. ^c^ Mann–Whitney test.

**Table 2:** DENV strains characterized in the present study.

Strain	Resident Area	Country	Collection Date	NS1 Ag	Anti-IgM	Anti-IgG	Serotype	Genotype	Accession Number	
									Envelope	Full Genome
DENVN19_075	ND	Vietnam	November 2019	POS	NEG	NEG	DENV-1	I	OQ832560	OQ832609
DENVN19_080	Hanoi	Vietnam	December 2019	POS	NEG	NEG	DENV-1	I	OQ832561	OQ832610
DENVN19_129	ND	Vietnam	October 2019	POS	NEG	NEG	DENV-1	I	ud	OQ832611
DENVN19_137	ND	Vietnam	October 2019	POS	NEG	NEG	DENV-1	I	OQ832562	OQ832612
DENVN20_020	Phu Tho	Vietnam	September 2020	POS	POS	NEG	DENV-1	I	OQ832563	ud
DENVN20_032	Hanoi	Vietnam	September 2020	POS	NEG	NEG	DENV-1	I	OQ832564	ud
DENVN20_059	Thanh Hoa	Vietnam	September 2020	POS	NEG	NEG	DENV-1	I	OQ832565	ud
DENVN20_066	Hanoi	Vietnam	October 2020	POS	NEG	NEG	DENV-1	I	OQ832566	ud
DENVN20_111	Hanoi	Vietnam	September 2020	POS	NEG	NEG	DENV-1	I	OQ832567	OQ832613
DENVN20_112	Hanoi	Vietnam	September 2020	POS	NEG	NEG	DENV-1	I	OQ832568	OQ832614
DENVN20_120	Hanoi	Vietnam	September 2020	POS	NEG	NEG	DENV-1	I	OQ832569	OQ832615
DENVN20_124	Hanoi	Vietnam	September 2020	POS	NEG	NEG	DENV-1	I	OQ832570	ud
DENVN20_226	Hanoi	Vietnam	October 2020	POS	NEG	NEG	DENV-1	I	OQ832571	ud
DENVN19_004	ND	Vietnam	October 2019	POS	NEG	NEG	DENV-2	Cosmopolitan	OQ832572	OQ832616
DENVN19_006	ND	Vietnam	October 2019	POS	NEG	NEG	DENV-2	Cosmopolitan	OQ832573	ud
DENVN19_010	ND	Vietnam	October 2019	POS	NEG	NEG	DENV-2	Asian-I	OQ832574	OQ832617
DENVN19_011	ND	Vietnam	October 2019	POS	NEG	NEG	DENV-2	Cosmopolitan	OQ832575	OQ832618
DENVN19_013	ND	Vietnam	October 2019	POS	NEG	NEG	DENV-2	Cosmopolitan	OQ832576	OQ832619
DENVN19_015	ND	Vietnam	October 2019	POS	NEG	NEG	DENV-2	Cosmopolitan	OQ832577	OQ832620
DENVN19_078	ND	Vietnam	December 2019	POS	NEG	NEG	DENV-2	Cosmopolitan	OQ832578	OQ832621
DENVN19_089	Hanoi	Vietnam	December 2019	POS	NEG	NEG	DENV-2	Cosmopolitan	OQ832579	OQ832622
DENVN19_140	ND	Vietnam	October 2019	POS	NEG	NEG	DENV-2	Asian-I	OQ832580	ud
DENVN19_142	ND	Vietnam	October 2019	POS	NEG	NEG	DENV-2	Cosmopolitan	OQ832581	ud
DENVN19_143	ND	Vietnam	October 2019	POS	NEG	NEG	DENV-2	Asian-I	OQ832582	OQ832623
DENVN19_144	ND	Vietnam	October 2019	POS	NEG	NEG	DENV-2	Cosmopolitan	OQ832583	ud
DENVN20_019	Hanoi	Vietnam	September 2020	POS	NEG	NEG	DENV-2	Cosmopolitan	OQ832584	ud
DENVN20_021	Hanoi	Vietnam	September 2020	POS	NEG	NEG	DENV-2	Cosmopolitan	OQ832585	ud
DENVN20_049	Hanoi	Vietnam	September 2020	POS	NEG	NEG	DENV-2	Cosmopolitan	OQ832586	ud
DENVN20_074	Hanoi	Vietnam	October 2020	POS	NEG	NEG	DENV-2	Cosmopolitan	OQ832587	ud
DENVN20_106	Hanoi	Vietnam	September 2020	POS	NEG	NEG	DENV-2	Cosmopolitan	OQ832588	ud
DENVN20_107	Hanoi	Vietnam	September 2020	POS	NEG	NEG	DENV-2	Cosmopolitan	OQ832589	OQ832624
DENVN20_113	Hanoi	Vietnam	September 2020	POS	NEG	NEG	DENV-2	Cosmopolitan	OQ832590	OQ832625
DENVN20_118	Hanoi	Vietnam	September 2020	POS	NEG	NEG	DENV-2	Asian-I	OQ832591	OQ832626
DENVN20_127	Hanoi	Vietnam	September 2020	POS	NEG	NEG	DENV-2	Asian-I	OQ832592	OQ832627
DENVN20_210	Hanoi	Vietnam	October 2020	POS	NEG	NEG	DENV-2	Cosmopolitan	OQ832593	ud
DENVN20_220	Hanoi	Vietnam	October 2020	POS	NEG	NEG	DENV-2	Cosmopolitan	OQ832594	ud

ND: no data; POS: positive; NEG: negative; ud: undetermined.

## Data Availability

The newly obtained sequences were deposited in GenBank with accession numbers OQ832560-OQ832594 and OQ832609-OQ832627. The viral sequences analyzed in the present study were retrieved using GenBank and BV-BRC and are listed in Table S2.

## Supplementary Materials

The following supporting information can be downloaded at https://www.mdpi.com/article/10.3390/microorganisms11051267/s1. Table S1: PCR and sequencing primers targeting the envelope gene region; Table S2: Sequences analyzed in the present study; Table S3: The model-fit comparison of the log marginal likelihood estimation (MLE) using path sampling (PS) and stepping-stone sampling (SS); Figure S1: Temporal signal analysis of regression of root-to-tip divergence against date; Figure S2: Phylogenetic tree of Vietnam strains from 1988 to 2022. The envelope sequences of 760 Vietnam DENV-2 strains from 1988 to 2022 and DENV-2 genotype reference strains were constructed for the maximum-likelihood tree under TIM2 + F + I + G4. The DENV-2 genotypes and the Bootstrap (>80%) are indicated at the adjacent branch. The tree branch is colored corresponding to genotypes. Vietnam taxa from a public database and obtained in the present study are labeled in blue and red, respectively.
