# Supplementary material for: Molecular Characterization of Dengue Virus Strains from the 2019–2020 Epidemic in Hanoi, Vietnam
Source: Microorganisms. 2023 May 11;11(5):1267. doi: 10.3390/microorganisms11051267 (PMC10222920; doi:10.3390/microorganisms11051267)

Table S1 PCR and sequencing primers targeting envelope gene region.

Target	Application	Primer	Sequence 5'-3'	Size (bp)	Annealing Temp
DENV-1 First product	1°PCR	D1-762F	CGTGGATGTCYTCTGAAGG	957	50 °C
		D1-1719R	GTGTGCATTGCTCCTTCTTG		
	2°PCR and Sequencing	D1-820F	GAGACACCCAGGATTCACG	899	55 °C
		D1-1719R	GTGTGCATTGCTCCTTCTTG		
DENV-1 Second product	1°PCR	D1-1643F	CTGGTGACATTTAAGACAGCT	916	50 °C
		D1-2559R	CCRATGGCYGCTGAYAGTCT		
	2°PCR and Sequencing	D1-1643F	CTGGTGACATTTAAGACAGCT	876	55 °C
		D1-2516R	TGTATTGCTCTGTCCAAGTG		
DENV-2 First product	1°PCR	D2-618F	ACCAGAAGACATAGATTGTTGGTGC	977	50 °C
		D2-1595R	CATGGTAAYGGCAGGTCT		
	2°PCR and Sequencing	D2-865Fx	ACCATAGGRACRACAYATTTCC	730	55 °C
		D2-1595R	CATGGTAAYGGCAGGTCT		
DENV-2 Second product	1°PCR	D2-1577Fx	TAGACCTGCCRTTACCATG	897	50 °C
		D2-2474R	CCACTGCCACATTTCAGTTC		
	2°PCR and Sequencing	D2-1577Fx	TAGACCTGCCRTTACCATG	888	55 °C
		D2-2465R	CATTTCAGTTCTTTRTTYTTCCAGC		

Table S2. Sequences analyzed in the present study.

Analysis	Serotype	Genotype	Accession number	Country	Collection year
DENV-1					
	1	I	AB074760	Japan	1943
	1	II	AF180817	Thailand	1964
	1	I	AF350498	China	1980
	1	IV	AF425611.1	Australia	1983
	1	IV	AF425627.1	Philippines	1974
	1	V	AY732476	Thailand	1980
	1	I	AY732481	Thailand	1982
	1	I	AY732483	Thailand	1981
	1	IV	D00502.1	Thailand	1980
	1	III	EF457905	Malaysia	1972
	1	I	EU848545.1	Hawaii	1944
	1	I	JN415487.1	Vietnam	2010
	1	V	JQ922544.1	India	1963
	1	II	JQ922547.1	Thailand	1960
	1	I	JX093667.1	Vietnam	2011
	1	I	JX093678.1	Vietnam	2011
	1	I	JX093680.1	Vietnam	2011
	1	I	JX093687.1	Vietnam	2011
	1	I	JX093690.1	Vietnam	2011
	1	I	JX093710.1	Vietnam	2011
	1	I	KC316022.1	NewCaledonia	2012
	1	I	KC741439.1	NewCaledonia	2012
	1	I	KC741442.1	NewCaledonia	2012
	1	I	KJ806952.1	Singapore	2013
	1	I	KT825033.1	Vietnam	2014
	1	I	KU570095.1	China	2012
	1	I	KY818248.1	Vietnam	2012
	1	I	KY818250.1	Vietnam	2012
	1	I	KY971714.1	Vietnam	2012
	1	I	KY971719.1	Vietnam	2015
	1	I	LC428061.1	Vietnam	2017
	1	I	LC428063.1	Vietnam	2017
	1	I	LC428064.1	Vietnam	2017
	1	I	LC428065.1	Vietnam	2017
	1	I	LC428067.1	Vietnam	2017
	1	I	LC428072.1	Vietnam	2017
	1	I	LC428075.1	Vietnam	2017
	1	I	LC428076.1	Vietnam	2017
	1	I	LC428077.1	Vietnam	2017
	1	I	MF033254.1	Singapore	2016
	1	I	MG738066.1	China	2017
	1	I	MG840558.1	Vietnam	2015
	1	I	MG894700.1	Vietnam	2012
	1	I	MG894867.1	Cambodia	2015
	1	I	MG894885.1	Vietnam	2015
	1	I	MG894893.1	Vietnam	2015
	1	I	MG894918.1	Vietnam	2016
	1	I	MG894943.1	Vietnam	2016
	1	I	MG894963.1	Vietnam	2016
	1	I	MG894965.1	Vietnam	2016
	1	I	MG894966.1	Vietnam	2016
	1	I	MH807161.1	FrenchPolynesia	2015
	1	I	MN628211.1	Laos	2015
	1	I	MN628212.1	Laos	2015
	1	I	MN628218.1	Laos	2015
	1	I	MN628230.1	Laos	2015
	1	I	MN912109.1	Vietnam	2017
	1	I	MN912110.1	Vietnam	2017
	1	I	MN912111.1	Vietnam	2017
	1	I	MN912112.1	Vietnam	2017
	1	I	MN912115.1	Vietnam	2017
	1	I	MN912117.1	Vietnam	2017
	1	I	MN912119.1	Vietnam	2017
	1	I	MN912130.1	Vietnam	2017
	1	I	MN912224.1	Vietnam	2017
	1	I	MN912225.1	Vietnam	2017
	1	I	MN912231.1	Vietnam	2017
	1	I	MN912233.1	Vietnam	2017
	1	I	MN912236.1	Vietnam	2017
	1	I	MN912240.1	Vietnam	2017
	1	I	MN912242.1	Vietnam	2017
	1	I	MN912244.1	Vietnam	2017
	1	I	MN912246.1	Vietnam	2017
	1	I	MN912247.1	Vietnam	2017
	1	I	MN912248.1	Vietnam	2017
	1	I	MN921292.1	China	2019
	1	I	MT856286.1	China	2017
	1	I	MW265687.1	NewCaledonia	2012
	1	I	MW265698.1	NewCaledonia	2013
	1	I	MW559393.1	Laos	2015
	1	I	MZ520699.1	Vietnam	2018
	1	I	MZ618896.1	NewCaledonia	2014
	1	I	MZ618987.1	Thailand	2019
	1	I	ON845601.1	Vietnam	2021
	1	I	ON911789.1	Vietnam	2019
	1	I	OP090342.1	Vietnam	2022
	1	I	OP090343.1	Vietnam	2022
	1	I	OP090344.1	Vietnam	2020
	1	I	OP090351.1	Vietnam	2020

Analysis	Serotype	Genotype	Accession number	Country	Collection year	
	1	I	OQ832560	Vietnam	2019	DENVN19_075
	1	I	OQ832561	Vietnam	2019	DENVN19_080
	1	I	OQ832562	Vietnam	2019	DENVN19_137
	1	I	OQ832563	Vietnam	2020	DENVN20_020
	1	I	OQ832564	Vietnam	2020	DENVN20_032
	1	I	OQ832565	Vietnam	2020	DENVN20_059
	1	I	OQ832566	Vietnam	2020	DENVN20_066
	1	I	OQ832567	Vietnam	2020	DENVN20_111
	1	I	OQ832568	Vietnam	2020	DENVN20_112
	1	I	OQ832569	Vietnam	2020	DENVN20_120
	1	I	OQ832570	Vietnam	2020	DENVN20_124
	1	I	OQ832571	Vietnam	2020	DENVN20_226
**DENV-2**						
	2	Asian-II	AF038403	NewGuinea	1944	
	2	Cosmopolitan	AF231716	Malaysia	1969	
	2	Asian-American	AY484607.3	PuertoRico	1988	
	2	Cosmopolitan	DQ518636	Malaysia	2004	
	2	Asian-American	DQ518639	Vietnam	2005	
	2	Asian-I	DQ518648	Vietnam	2005	
	2	Cosmopolitan	EU056810	BurkinaFaso	1983	
	2	Cosmopolitan	EU448428	Vietnam	2007	
	2	Asian-I	EU482472	Vietnam	2004	
	2	Cosmopolitan	EU482640	Vietnam	2006	
	2	Asian-I	EU482657	Vietnam	2006	
	2	Cosmopolitan	EU482672	Vietnam	2006	
	2	Asian-I	EU482784	Vietnam	2003	
	2	Asian-I	EU482786	Vietnam	2003	
	2	Asian-I	FJ410221	Vietnam	2007	
	2	Cosmopolitan	FJ538920	India	1974	
	2	Asian-American	FJ639697	Cambodia	2001	
	2	Asian-American	FJ639698	Cambodia	2002	
	2	Asian-American	FJ639703	Cambodia	2003	
	2	Asian-American	FM210202	Vietnam	2004	
	2	Asian-I	FM210207	Vietnam	2005	
	2	Asian-American	FM210213	Vietnam	2005	
	2	Asian-American	FM210217	Vietnam	1999	
	2	Asian-American	FM210218	Vietnam	2002	
	2	Asian-I	FM210240	Vietnam	2004	
	2	Cosmopolitan	GQ398258	Indonesia	1975	
	2	Cosmopolitan	GQ398261	Indonesia	1976	
	2	Cosmopolitan	GQ398263	Indonesia	1975	
	2	Asian-II	GQ398268	Indonesia	1975	
	2	American	GQ868592	Colombia	1986	
	2	Asian-I	GU211738	Vietnam	2004	
	2	Asian-American	GU211743	Vietnam	2003	
	2	Asian-American	GU211751	Vietnam	2006	
	2	Asian-American	GU211762	Vietnam	2006	
	2	Asian-American	GU434154	Vietnam	2001	
	2	Asian-American	GU434156	Vietnam	2003	
	2	Asian-I	GU434158	Vietnam	2004	
	2	Asian-American	GU434159	Vietnam	2004	
	2	Cosmopolitan	GU908496	Vietnam	2009	
	2	Asian-I	GU908498	Vietnam	2009	
	2	Asian-I	HQ588132	Vietnam	2007	
	2	Asian-I	HQ588140	Vietnam	2006	
	2	Asian-I	JN376791	Vietnam	2008	
	2	Asian-American	JN819418	Vietnam	1988	
	2	Cosmopolitan	JN851113	Singapore	2006	
	2	Asian-I	JX093591	Vietnam	2011	
	2	Cosmopolitan	JX093594	Vietnam	2011	
	2	Cosmopolitan	JX093609	Vietnam	2011	
	2	Asian-I	JX093638	Vietnam	2011	
	2	Asian-I	JX093643	Vietnam	2011	
	2	Asian-American	JX649147	Vietnam	1995	
	2	Asian-American	KF955363	PuertoRico	1986	
	2	Cosmopolitan	KJ806910	Malaysia	2013	
	2	Asian-I	KP671756	Vietnam	2012	
	2	Asian-I	KP769810	Vietnam	2012	
	2	Asian-I	KT175128	Vietnam	2012	
	2	Cosmopolitan	KT175129	Vietnam	2014	
	2	Cosmopolitan	KT781567	Vietnam	2014	
	2	Cosmopolitan	KT781568	Indonesia	2015	
	2	Cosmopolitan	KX270815	China	2010	
	2	Asian-I	KY495819	Cambodia	2015	
	2	Cosmopolitan	KY627762	BurkinaFaso	2016	
	2	Cosmopolitan	KY709187	Indonesia	2015	
	2	Cosmopolitan	KY851389	Indonesia	2012	
	2	Asian-I	KY851493	Vietnam	2012	
	2	Cosmopolitan	KY971720	Vietnam	2015	
	2	Cosmopolitan	KY971721	Vietnam	2015	
	2	Cosmopolitan	KY971722	Vietnam	2015	
	2	Cosmopolitan	KY971723	Vietnam	2015	
	2	Cosmopolitan	KY971724	Vietnam	2015	
	2	Cosmopolitan	KY971725	Vietnam	2015	
	2	Cosmopolitan	KY971726	Vietnam	2015	
	2	Cosmopolitan	KY971727	Vietnam	2015	
	2	Cosmopolitan	KY971728	Vietnam	2015	
	2	Cosmopolitan	KY971729	Vietnam	2015	
	2	Cosmopolitan	KY971730	Vietnam	2015	
	2	Cosmopolitan	KY971731	Vietnam	2015	
	2	Cosmopolitan	KY971732	Vietnam	2015	
	2	Cosmopolitan	KY971733	Vietnam	2015	
	2	Cosmopolitan	KY971734	Vietnam	2015	
	2	Cosmopolitan	KY971735	Vietnam	2015	

Analysis	Serotype	Genotype	Accession number	Country	Collection year
	2	Cosmopolitan	KY971736	Vietnam	2015
	2	Cosmopolitan	KY971737	Vietnam	2015
	2	Cosmopolitan	KY971738	Vietnam	2015
	2	Cosmopolitan	KY971739	Vietnam	2015
	2	Cosmopolitan	KY971740	Vietnam	2015
	2	Cosmopolitan	KY971741	Vietnam	2015
	2	Cosmopolitan	LC410190	Thailand	2016
	2	Cosmopolitan	LC410191	Thailand	2017
	2	Asian-I	LC553001	Japan	2019
	2	Cosmopolitan	MF156237	China	2015
	2	Cosmopolitan	MF940248	China	2015
	2	Asian-I	MG737984	Vietnam_China	2015
	2	Asian-I	MG894978	Vietnam	2011
	2	Asian-I	MG894980	Vietnam	2011
	2	Asian-I	MG894983	Vietnam	2011
	2	Cosmopolitan	MG894991	Vietnam	2011
	2	Asian-I	MG895013	Vietnam	2012
	2	Asian-I	MG895014	Vietnam	2012
	2	Asian-I	MG895017	Vietnam	2013
	2	Asian-I	MG895033	Vietnam	2013
	2	Cosmopolitan	MG895039	Vietnam	2013
	2	Cosmopolitan	MG895076	Malaysia	2015
	2	Asian-I	MG895101	Vietnam	2015
	2	Asian-I	MG895102	Vietnam	2015
	2	Asian-I	MG895109	Vietnam	2015
	2	Cosmopolitan	MG895118	Vietnam	2015
	2	Cosmopolitan	MG895119	Vietnam	2015
	2	Cosmopolitan	MG895120	Vietnam	2015
	2	Cosmopolitan	MG895121	Vietnam	2015
	2	Asian-I	MG895129	Vietnam	2015
	2	Asian-I	MG895130	Vietnam	2015
	2	Cosmopolitan	MG895150	Thailand	2016
	2	Asian-I	MG895161	Laos	2016
	2	Asian-I	MG895167	Vietnam	2016
	2	Asian-I	MH010611	China	2017
	2	Cosmopolitan	MH173164	Indonesia	2016
	2	Cosmopolitan	MH822942	India	2014
	2	Cosmopolitan	MH827525	China	2014
	2	Cosmopolitan	MK564479	China	2016
	2	Cosmopolitan	MK578531	China	2016
	2	Cosmopolitan	MK783199	China	2018
	2	Cosmopolitan	MK858111	India	2016
	2	Cosmopolitan	MN444605	Laos	2015
	2	Cosmopolitan	MN444606	Laos	2016
	2	Cosmopolitan	MN444609	Laos	2017
	2	Asian-I	MN444611	Laos	2017
	2	Cosmopolitan	MN444614	Laos	2018
	2	Cosmopolitan	MN444618	Laos	2017
	2	Asian-I	MN923114	Cambodia_China	2019
	2	Cosmopolitan	MN923116	China	2019
	2	Cosmopolitan	MN952967	China	2015
	2	Cosmopolitan	MN955677	Thailand	2018
	2	Cosmopolitan	MN982889	Indonesia	2019
	2	Cosmopolitan	MN982899	Australia	2019
	2	Cosmopolitan	MT856323	China	2019
	2	Asian-I	MT856336	China	2019
	2	Cosmopolitan	MT982917	Mali	2017
	2	Cosmopolitan	MW288029	Senegal	2018
	2	Cosmopolitan	MW512413	Singapore	2014
	2	Cosmopolitan	MW512448	Singapore	2015
	2	Cosmopolitan	MW512451	Singapore	2016
	2	Cosmopolitan	MW512466	Singapore	2016
	2	Cosmopolitan	MW512468	Singapore	2017
	2	Cosmopolitan	MW512469	Singapore	2017
	2	Cosmopolitan	MW945435	Vietnam	2006
	2	Cosmopolitan	MW946478	India	1974
	2	Cosmopolitan	MZ277506	India	2019
	2	Cosmopolitan	MZ277514	India	2018
	2	Asian-I	MZ636761	Thailand	2018
	2	Cosmopolitan	MZ636781	Thailand	2019
	2	Cosmopolitan	MZ636804	Thailand	2019
	2	Asian-I	NC_001474	Thailand	1964
	2	Cosmopolitan	OK180527	Indonesia	2016
	2	Cosmopolitan	OL412740	Cambodia	2019
	2	Asian-I	OL414743	Cambodia	2019
	2	Cosmopolitan	OL414746	Cambodia	2020
	2	Asian-I	OL414761	Cambodia	2019
	2	Cosmopolitan	OM317566	Cameroon	2020
	2	Cosmopolitan	OP090388	Vietnam	2022
	2	Cosmopolitan	OP090389	Vietnam	2022
	2	Cosmopolitan	OP090390	Vietnam	2022
	2	Cosmopolitan	OP090391	Vietnam	2022
	2	Cosmopolitan	OP090392	Vietnam	2022
	2	Cosmopolitan	OP090393	Vietnam	2022
	2	Asian-I	OP090394	Vietnam	2022
	2	Asian-I	OP090395	Vietnam	2021
	2	Asian-I	OP090396	Vietnam	2022
	2	Asian-I	OP090397	Vietnam	2020
	2	Cosmopolitan	OP410989	Singapore	2007
	2	Cosmopolitan	OP458511	Vietnam	2019
	2	Cosmopolitan	OP458512	Vietnam	2020
	2	Asian-I	OP458513	Vietnam	2019
	2	Cosmopolitan	OP458514	Vietnam	2020
	2	Cosmopolitan	OP458515	Vietnam	2021

Analysis	Serotype	Genotype	Accession number	Country	Collection year	
	2	Cosmopolitan	OP458516	Vietnam	2020	
	2	Cosmopolitan	OP458517	Vietnam	2019	
	2	Cosmopolitan	OP684209	China	2019	
	2	Cosmopolitan	OP895918	Cambodia	2019	
	2	Cosmopolitan	OP984834	Vietnam	2022	
	2	Asian-I	OQ028205	Vietnam	2017	
	2	Cosmopolitan	OQ028206	Vietnam	2018	
	2	Cosmopolitan	OQ028207	Vietnam	2019	
	2	Cosmopolitan	OQ028208	Vietnam	2019	
	2	Cosmopolitan	OQ028209	Vietnam	2020	
	2	Cosmopolitan	OQ028210	Vietnam	2020	
	2	Cosmopolitan	OQ028211	Vietnam	2020	
	2	Cosmopolitan	OQ028212	Vietnam	2020	
	2	Cosmopolitan	OQ028213	Vietnam	2019	
	2	Cosmopolitan	OQ028214	Vietnam	2019	
	2	Asian-I	OQ028215	Vietnam	2019	
	2	Cosmopolitan	OQ028216	Vietnam	2022	
	2	Cosmopolitan	OQ028217	Vietnam	2019	
	2	Asian-I	OQ028218	Vietnam	2019	
	2	Asian-I	OQ028219	Vietnam	2020	
	2	Cosmopolitan	OQ028220	Vietnam	2019	
	2	Asian-I	OQ028221	Vietnam	2019	
	2	Asian-I	OQ028222	Vietnam	2019	
	2	Asian-I	OQ028223	Vietnam	2019	
	2	Asian-I	OQ028224	Vietnam	2019	
	2	Cosmopolitan	OQ028225	Vietnam	2020	
	2	Asian-I	OQ028226	Vietnam	2019	
	2	Cosmopolitan	OQ028227	Vietnam	2019	
	2	Asian-I	OQ028228	Vietnam	2019	
	2	Asian-I	OQ028229	Vietnam	2017	
	2	Cosmopolitan	OQ028230	Vietnam	2020	
	2	Asian-I	OQ028231	Vietnam	2018	
	2	Cosmopolitan	OQ028232	Vietnam	2020	
	2	Cosmopolitan	OQ426757	Vietnam	2018	
	2	Cosmopolitan	OQ426766	Vietnam	2019	
	2	Cosmopolitan	OQ426773	Vietnam	2018	
	2	Cosmopolitan	OQ426782	Vietnam	2018	
	2	Asian-I	OQ426797	Vietnam	2018	
	2	Asian-I	OQ426819	Vietnam	2018	
	2	Asian-I	OQ426843	Vietnam	2018	
	2	Cosmopolitan	OQ426847	Vietnam	2018	
	2	Asian-I	OQ426879	Vietnam	2019	
	2	Asian-I	OQ426905	Vietnam	2019	
	2	Asian-I	OQ426907	Vietnam	2017	
	2	Asian-I	OQ426912	Vietnam	2018	
	2	Cosmopolitan	OQ426916	Vietnam	2018	
	2	Cosmopolitan	OQ426918	Vietnam	2018	
	2	Asian-I	OQ426919	Vietnam	2018	
	2	Asian-I	OQ426956	Vietnam	2018	
	2	Cosmopolitan	OQ832572	Vietnam	2019	DENVN19_004
	2	Cosmopolitan	OQ832573	Vietnam	2019	DENVN19_006
	2	Asian-I	OQ832574	Vietnam	2019	DENVN19_010
	2	Cosmopolitan	OQ832575	Vietnam	2019	DENVN19_011
	2	Cosmopolitan	OQ832576	Vietnam	2019	DENVN19_013
	2	Cosmopolitan	OQ832577	Vietnam	2019	DENVN19_015
	2	Cosmopolitan	OQ832578	Vietnam	2019	DENVN19_078
	2	Cosmopolitan	OQ832579	Vietnam	2019	DENVN19_089
	2	Asian-I	OQ832580	Vietnam	2019	DENVN19_140
	2	Cosmopolitan	OQ832581	Vietnam	2019	DENVN19_142
	2	Asian-I	OQ832582	Vietnam	2019	DENVN19_143
	2	Cosmopolitan	OQ832583	Vietnam	2019	DENVN19_144
	2	Cosmopolitan	OQ832584	Vietnam	2020	DENVN20_019
	2	Cosmopolitan	OQ832585	Vietnam	2020	DENVN20_021
	2	Cosmopolitan	OQ832586	Vietnam	2020	DENVN20_049
	2	Cosmopolitan	OQ832587	Vietnam	2020	DENVN20_074
	2	Cosmopolitan	OQ832588	Vietnam	2020	DENVN20_106
	2	Cosmopolitan	OQ832589	Vietnam	2020	DENVN20_107
	2	Cosmopolitan	OQ832590	Vietnam	2020	DENVN20_113
	2	Asian-I	OQ832591	Vietnam	2020	DENVN20_118
	2	Asian-I	OQ832592	Vietnam	2020	DENVN20_127
	2	Cosmopolitan	OQ832593	Vietnam	2020	DENVN20_210
	2	Cosmopolitan	OQ832594	Vietnam	2020	DENVN20_220
**DENV-2 Cosmopolitan**						
	2	Cosmopolitan	AF231716	Malaysia	1969	
	2	Cosmopolitan	AY858035	Indonesia	2004	
	2	Cosmopolitan	DQ518636	Malaysia	2004	
	2	Cosmopolitan	EU056810	BurkinaFaso	1983	
	2	Cosmopolitan	EU448428	Vietnam	2007	
	2	Cosmopolitan	EU482640	Vietnam	2006	
	2	Cosmopolitan	EU482672	Vietnam	2006	
	2	Cosmopolitan	FJ538920	India	1974	
	2	Cosmopolitan	GQ398258	Indonesia	1975	
	2	Cosmopolitan	GQ398261	Indonesia	1976	
	2	Cosmopolitan	GQ398263	Indonesia	1975	
	2	Cosmopolitan	GQ398264	Indonesia	1976	
	2	Cosmopolitan	GU908496	Vietnam	2009	
	2	Cosmopolitan	JF327392	Singapore	2009	
	2	Cosmopolitan	JF968007	Indonesia	2010	
	2	Cosmopolitan	JN851113	Singapore	2006	
	2	Cosmopolitan	JX093609	Vietnam	2011	
	2	Cosmopolitan	KC131142	China	2012	
	2	Cosmopolitan	KC762667	Indonesia	2008	
	2	Cosmopolitan	KC762671	Indonesia	2008	
	2	Cosmopolitan	KF744397	Philippines	2001	

Analysis	Serotype	Genotype	Accession number	Country	Collection year
	2	Cosmopolitan	KJ806811	Singapore	2014
	2	Cosmopolitan	KJ806891	Malaysia	2013
	2	Cosmopolitan	KJ806910	Malaysia	2013
	2	Cosmopolitan	KT175129	Vietnam	2014
	2	Cosmopolitan	KT781567	Vietnam	2014
	2	Cosmopolitan	KU517846	Indonesia	2014
	2	Cosmopolitan	KX270815	China	2010
	2	Cosmopolitan	KX372564	Australia	2015
	2	Cosmopolitan	KX380815	Singapore	2012
	2	Cosmopolitan	KX452030	Malaysia	2014
	2	Cosmopolitan	KX452042	Malaysia	2014
	2	Cosmopolitan	KX577683	China	2015
	2	Cosmopolitan	KY627762	BurkinaFaso	2016
	2	Cosmopolitan	KY709187	Indonesia	2015
	2	Cosmopolitan	KY851436	Philippines	2013
	2	Cosmopolitan	KY971720	Vietnam	2015
	2	Cosmopolitan	KY971721	Vietnam	2015
	2	Cosmopolitan	KY971722	Vietnam	2015
	2	Cosmopolitan	KY971723	Vietnam	2015
	2	Cosmopolitan	KY971724	Vietnam	2015
	2	Cosmopolitan	KY971725	Vietnam	2015
	2	Cosmopolitan	KY971726	Vietnam	2015
	2	Cosmopolitan	KY971727	Vietnam	2015
	2	Cosmopolitan	KY971728	Vietnam	2015
	2	Cosmopolitan	KY971729	Vietnam	2015
	2	Cosmopolitan	KY971730	Vietnam	2015
	2	Cosmopolitan	KY971731	Vietnam	2015
	2	Cosmopolitan	KY971732	Vietnam	2015
	2	Cosmopolitan	KY971733	Vietnam	2015
	2	Cosmopolitan	KY971734	Vietnam	2015
	2	Cosmopolitan	KY971735	Vietnam	2015
	2	Cosmopolitan	KY971736	Vietnam	2015
	2	Cosmopolitan	KY971737	Vietnam	2015
	2	Cosmopolitan	KY971738	Vietnam	2015
	2	Cosmopolitan	KY971739	Vietnam	2015
	2	Cosmopolitan	KY971740	Vietnam	2015
	2	Cosmopolitan	KY971741	Vietnam	2015
	2	Cosmopolitan	LC410190	Thailand	2016
	2	Cosmopolitan	LC410191	Thailand	2017
	2	Cosmopolitan	LC436669	Bangladesh	2017
	2	Cosmopolitan	LC436673	Bangladesh	2017
	2	Cosmopolitan	LC436674	Bangladesh	2017
	2	Cosmopolitan	MF940248	China	2015
	2	Cosmopolitan	MG840586	China	2016
	2	Cosmopolitan	MG840629	China	2017
	2	Cosmopolitan	MG894991	Vietnam	2011
	2	Cosmopolitan	MG895039	Vietnam	2013
	2	Cosmopolitan	MG895076	Malaysia	2015
	2	Cosmopolitan	MG895118	Vietnam	2015
	2	Cosmopolitan	MG895119	Vietnam	2015
	2	Cosmopolitan	MG895120	Vietnam	2015
	2	Cosmopolitan	MG895121	Vietnam	2015
	2	Cosmopolitan	MG895150	Thailand	2016
	2	Cosmopolitan	MH048672	Malaysia	2014
	2	Cosmopolitan	MH110564	China	2017
	2	Cosmopolitan	MH173164	Indonesia	2016
	2	Cosmopolitan	MH729974	China	2015
	2	Cosmopolitan	MH729981	China	2016
	2	Cosmopolitan	MH822942	India	2014
	2	Cosmopolitan	MH827525	China	2014
	2	Cosmopolitan	MH827548	Maldives	2017
	2	Cosmopolitan	MH827551	Maldives	2017
	2	Cosmopolitan	MK543448	China	2018
	2	Cosmopolitan	MK564479	China	2016
	2	Cosmopolitan	MK564480	China	2016
	2	Cosmopolitan	MK578531	China	2016
	2	Cosmopolitan	MK783199	China	2018
	2	Cosmopolitan	MK783201	China	2018
	2	Cosmopolitan	MK858111	India	2016
	2	Cosmopolitan	MN018343	China	2017
	2	Cosmopolitan	MN018353	China	2016
	2	Cosmopolitan	MN083229	SriLanka	2017
	2	Cosmopolitan	MN444605	Laos	2015
	2	Cosmopolitan	MN444606	Laos	2016
	2	Cosmopolitan	MN444609	Laos	2017
	2	Cosmopolitan	MN444613	Laos	2018
	2	Cosmopolitan	MN444614	Laos	2018
	2	Cosmopolitan	MN444615	Laos	2018
	2	Cosmopolitan	MN444616	Laos	2018
	2	Cosmopolitan	MN444618	Laos	2017
	2	Cosmopolitan	MN444622	Laos	2017
	2	Cosmopolitan	MN548844	Vanuatu	2019
	2	Cosmopolitan	MN548846	NewCaledonia	2019
	2	Cosmopolitan	MN548855	WallisFutuna	2019
	2	Cosmopolitan	MN577555	Kenya	2017
	2	Cosmopolitan	MN923116	China	2019
	2	Cosmopolitan	MN952967	China	2015
	2	Cosmopolitan	MN955677	Thailand	2018
	2	Cosmopolitan	MN982889	Indonesia	2019
	2	Cosmopolitan	MN982899	Australia	2019
	2	Cosmopolitan	MN982900	Australia	2019
	2	Cosmopolitan	MN982901	Australia	2019
	2	Cosmopolitan	MT006171	SriLanka	2018
	2	Cosmopolitan	MT006175	SriLanka	2018

Analysis	Serotype	Genotype	Accession number	Country	Collection year
	2	Cosmopolitan	MT799882	NewCaledonia	2020
	2	Cosmopolitan	MT856314	China	2017
	2	Cosmopolitan	MT856323	China	2019
	2	Cosmopolitan	MT921573	Australia	2004
	2	Cosmopolitan	MT982917	Mali	2017
	2	Cosmopolitan	MW288029	Senegal	2018
	2	Cosmopolitan	MW481670	Anglola	2019
	2	Cosmopolitan	MW481671	Anglola	2018
	2	Cosmopolitan	MW510042	Singapore	2018
	2	Cosmopolitan	MW510249	Singapore	2011
	2	Cosmopolitan	MW512369	Singapore	2011
	2	Cosmopolitan	MW512413	Singapore	2014
	2	Cosmopolitan	MW512418	Singapore	2014
	2	Cosmopolitan	MW512428	Singapore	2014
	2	Cosmopolitan	MW512448	Singapore	2015
	2	Cosmopolitan	MW512451	Singapore	2016
	2	Cosmopolitan	MW512452	Singapore	2016
	2	Cosmopolitan	MW512454	Singapore	2016
	2	Cosmopolitan	MW512462	Singapore	2016
	2	Cosmopolitan	MW512465	Singapore	2016
	2	Cosmopolitan	MW512466	Singapore	2016
	2	Cosmopolitan	MW512468	Singapore	2017
	2	Cosmopolitan	MW512469	Singapore	2017
	2	Cosmopolitan	MW512471	Singapore	2017
	2	Cosmopolitan	MW512473	Singapore	2017
	2	Cosmopolitan	MW512475	Singapore	2017
	2	Cosmopolitan	MW512484	Singapore	2018
	2	Cosmopolitan	MW585365	NewCaledonia	2017
	2	Cosmopolitan	MW721471	China	2014
	2	Cosmopolitan	MW730825	Nepal	2017
	2	Cosmopolitan	MW730831	Nepal	2017
	2	Cosmopolitan	MW945435	Vietnam	2006
	2	Cosmopolitan	MW946478	India	1974
	2	Cosmopolitan	MZ277459	India	2018
	2	Cosmopolitan	MZ277506	India	2019
	2	Cosmopolitan	MZ277514	India	2018
	2	Cosmopolitan	MZ277532	India	2018
	2	Cosmopolitan	MZ312931	India	2017
	2	Cosmopolitan	MZ636781	Thailand	2019
	2	Cosmopolitan	MZ636802	Thailand	2019
	2	Cosmopolitan	MZ636804	Thailand	2019
	2	Cosmopolitan	OK180527	Indonesia	2016
	2	Cosmopolitan	OL412740	Cambodia	2019
	2	Cosmopolitan	OL414746	Cambodia	2020
	2	Cosmopolitan	OL960215	Indonesia	2016
	2	Cosmopolitan	OL960221	Indonesia	2015
	2	Cosmopolitan	OM317565	Cameroon	2020
	2	Cosmopolitan	OM639981	India	2021
	2	Cosmopolitan	OM680963	India	2021
	2	Cosmopolitan	OM791800	Peru	2019
	2	Cosmopolitan	OM791801	Peru	2019
	2	Cosmopolitan	ON123631	Peru	2021
	2	Cosmopolitan	ON634745	Brazil	2022
	2	Cosmopolitan	ON634756	Brazil	2022
	2	Cosmopolitan	OP090388	Vietnam	2022
	2	Cosmopolitan	OP090389	Vietnam	2022
	2	Cosmopolitan	OP090390	Vietnam	2022
	2	Cosmopolitan	OP090391	Vietnam	2022
	2	Cosmopolitan	OP090392	Vietnam	2022
	2	Cosmopolitan	OP090393	Vietnam	2022
	2	Cosmopolitan	OP410989	Singapore	2007
	2	Cosmopolitan	OP458511	Vietnam	2019
	2	Cosmopolitan	OP458512	Vietnam	2020
	2	Cosmopolitan	OP458514	Vietnam	2020
	2	Cosmopolitan	OP458515	Vietnam	2021
	2	Cosmopolitan	OP458516	Vietnam	2020
	2	Cosmopolitan	OP458517	Vietnam	2019
	2	Cosmopolitan	OP684209	China	2019
	2	Cosmopolitan	OP684210	China	2019
	2	Cosmopolitan	OP811982	Pakistan	2022
	2	Cosmopolitan	OP895917	Maldives	2021
	2	Cosmopolitan	OP895918	Cambodia	2019
	2	Cosmopolitan	OP984834	Vietnam	2022
	2	Cosmopolitan	OQ028206	Vietnam	2018
	2	Cosmopolitan	OQ028207	Vietnam	2019
	2	Cosmopolitan	OQ028208	Vietnam	2019
	2	Cosmopolitan	OQ028209	Vietnam	2020
	2	Cosmopolitan	OQ028210	Vietnam	2020
	2	Cosmopolitan	OQ028211	Vietnam	2020
	2	Cosmopolitan	OQ028212	Vietnam	2020
	2	Cosmopolitan	OQ028213	Vietnam	2019
	2	Cosmopolitan	OQ028214	Vietnam	2019
	2	Cosmopolitan	OQ028216	Vietnam	2022
	2	Cosmopolitan	OQ028217	Vietnam	2019
	2	Cosmopolitan	OQ028220	Vietnam	2019
	2	Cosmopolitan	OQ028225	Vietnam	2020
	2	Cosmopolitan	OQ028227	Vietnam	2019
	2	Cosmopolitan	OQ028230	Vietnam	2020
	2	Cosmopolitan	OQ028232	Vietnam	2020
	2	Cosmopolitan	OQ426757	Vietnam	2018
	2	Cosmopolitan	OQ426766	Vietnam	2019
	2	Cosmopolitan	OQ426773	Vietnam	2018
	2	Cosmopolitan	OQ426782	Vietnam	2018
	2	Cosmopolitan	OQ426847	Vietnam	2018

Analysis	Serotype	Genotype	Accession number	Country	Collection year	
	2	Cosmopolitan	OQ426916	Vietnam	2018	
	2	Cosmopolitan	OQ426918	Vietnam	2018	
	2	Cosmopolitan	OQ832572	Vietnam	2019	DENVN19_004
	2	Cosmopolitan	OQ832573	Vietnam	2019	DENVN19_006
	2	Cosmopolitan	OQ832575	Vietnam	2019	DENVN19_011
	2	Cosmopolitan	OQ832576	Vietnam	2019	DENVN19_013
	2	Cosmopolitan	OQ832577	Vietnam	2019	DENVN19_015
	2	Cosmopolitan	OQ832578	Vietnam	2019	DENVN19_078
	2	Cosmopolitan	OQ832579	Vietnam	2019	DENVN19_089
	2	Cosmopolitan	OQ832581	Vietnam	2019	DENVN19_142
	2	Cosmopolitan	OQ832583	Vietnam	2019	DENVN19_144
	2	Cosmopolitan	OQ832584	Vietnam	2020	DENVN20_019
	2	Cosmopolitan	OQ832585	Vietnam	2020	DENVN20_021
	2	Cosmopolitan	OQ832586	Vietnam	2020	DENVN20_049
	2	Cosmopolitan	OQ832587	Vietnam	2020	DENVN20_074
	2	Cosmopolitan	OQ832588	Vietnam	2020	DENVN20_106
	2	Cosmopolitan	OQ832589	Vietnam	2020	DENVN20_107
	2	Cosmopolitan	OQ832590	Vietnam	2020	DENVN20_113
	2	Cosmopolitan	OQ832593	Vietnam	2020	DENVN20_210
	2	Cosmopolitan	OQ832594	Vietnam	2020	DENVN20_220
**Amino acid polymorphisms**						
	1	I	OQ832609	Vietnam	2019	DENVN19_075
	1	I	OQ832610	Vietnam	2019	DENVN19_080
	1	I	OQ832611	Vietnam	2019	DENVN19_129
	1	I	OQ832612	Vietnam	2019	DENVN19_137
	1	I	OQ832613	Vietnam	2020	DENVN20_111
	1	I	OQ832614	Vietnam	2020	DENVN20_112
	1	I	OQ832615	Vietnam	2020	DENVN20_120
	2	Cosmopolitan	OQ832616	Vietnam	2019	DENVN19_004
	2	Asian-I	OQ832617	Vietnam	2019	DENVN19_010
	2	Cosmopolitan	OQ832618	Vietnam	2019	DENVN19_011
	2	Cosmopolitan	OQ832619	Vietnam	2019	DENVN19_013
	2	Cosmopolitan	OQ832620	Vietnam	2019	DENVN19_015
	2	Cosmopolitan	OQ832621	Vietnam	2019	DENVN19_078
	2	Cosmopolitan	OQ832622	Vietnam	2019	DENVN19_089
	2	Asian-I	OQ832623	Vietnam	2019	DENVN19_143
	2	Cosmopolitan	OQ832624	Vietnam	2020	DENVN20_107
	2	Cosmopolitan	OQ832625	Vietnam	2020	DENVN20_113
	2	Asian-I	OQ832626	Vietnam	2020	DENVN20_118
	2	Asian-I	OQ832627	Vietnam	2020	DENVN20_127

Table S3. The model-fit comparison of the log marginal likelihood estimation (MLE) using path sampling (PS) and stepping-stone sampling (SS).

Clock Model	log marginal Likelihood	
	PS	SS
Strict clock	-9966.96	-9971.40
Uncorrelated relaxed clock	-9970.85	-9973.69

Figure S1. Temporal signal analysis of regression of root-to-tip divergence against date.

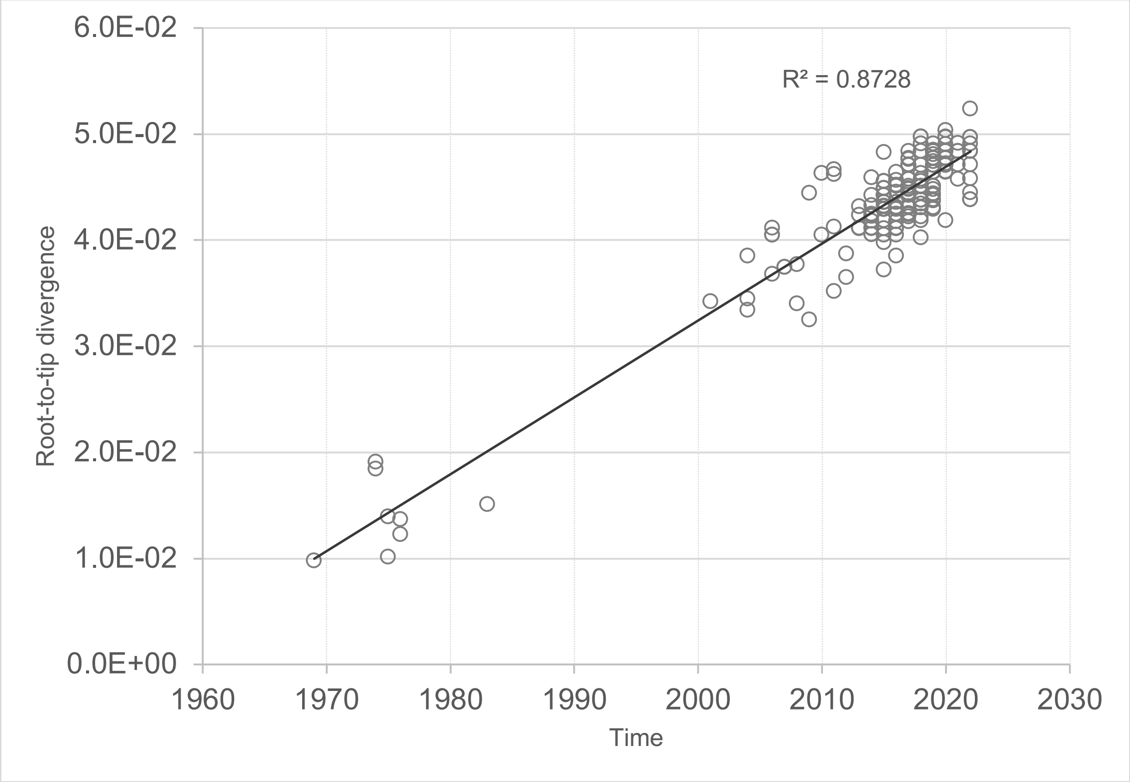

Figure S2. Phylogenetic tree of Vietnam strains from 1988-2022. The envelope sequences of 760 Vietnam DENV-2 strains from 1988-2022 and DENV-2 genotype reference strains were constructed for the Maximum Likelihood tree under TIM2+F+I+G4. The DENV-2 genotypes and the Bootstrap (>80%) are indicated at the adjacent branch. The tree branch is colored corresponding to geno-types. Vietnam taxa from a public database and obtained in the present study are labeled in blue and red, respectively.

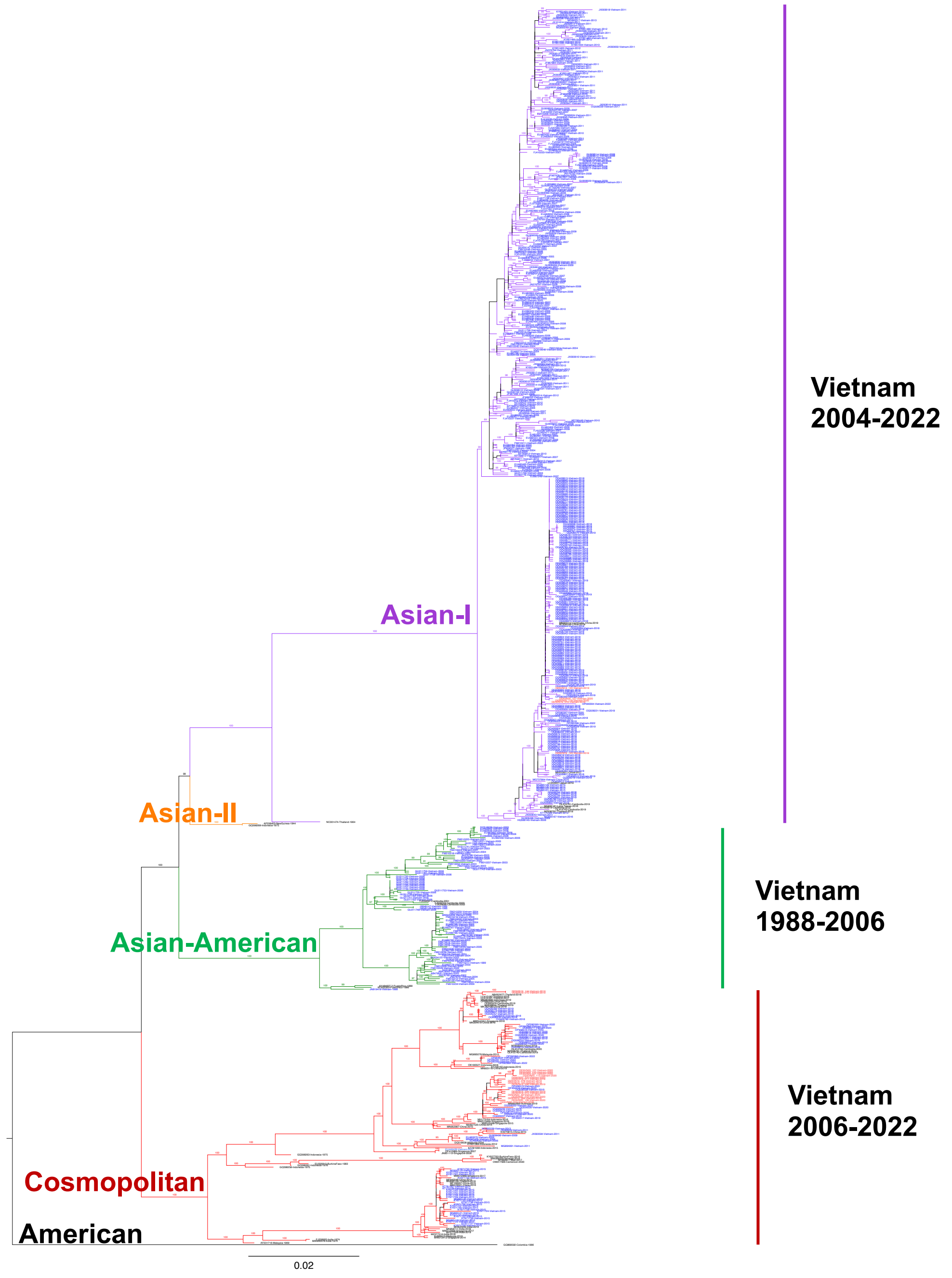

Supplement: Supplementary file 1 [file microorganisms-11-01267-s001.zip › microorganisms-2381092-supplementary.pdf]
